# The career paths of researchers in long-term employment on short-term contracts: Case study from a UK university

**DOI:** 10.1371/journal.pone.0274486

**Published:** 2022-09-09

**Authors:** Cecile B. Menard, Sara Shinton

**Affiliations:** 1 Institute for Academic Development, University of Edinburgh, Edinburgh, United Kingdom; 2 School of Geosciences, University of Edinburgh, Edinburgh, United Kingdom; University of Siena, Italy, ITALY

## Abstract

The career stage between PhD and lectureship, conventionally called “postdoctoral”, has traditionally been seen as transitional. However, with an estimated one third of university researchers in the United Kingdom having been employed on temporary contracts for more than 10 years, the transitional nature of this career stage is questionable. Despite so many research staff being in long-term employment on short-term contracts, the lack of visibility of this population, which does not have a legitimate place within the current academic career structure, means that we do not know how deliberate or accidental their career choices are. Based on semi-structured interviews with long-term researchers (LTRS) at one university in the United Kingdom, this is the first study to investigate the personal and professional circumstances behind the career path of long-term researchers on temporary contracts. Three categories of LTRS were identified: 1) the candidate, who wants to follow the traditional academic career pathway and to secure a lectureship 2) the accidental long-term researcher, who did not or could not plan their career path 3) the career researcher, who sustains a research-only career despite the precarity of such positions. Most participants had belonged successively to two categories. Some obstacles to career progression transcended the categories: inequal access to opportunities for developing one’s teaching portfolio, poor or lack of managerial support, the perceived prestige or lack thereof of one’s field, and bullying and discrimination. We argue that short-termism and lack of visibility play down considerably the contribution of long-term researchers to the financial and academic success of research institutions. We also argue that traditional–but still in place–structures in academia are ill adapted to the contemporary demographics and practices of the research community; we recommend that future studies involve HE sector stakeholders to review and to reform the academic career structure.

## 1. Introduction

“*It should be clear to everybody*, *including postdocs and supervisors*, *that [postdoctoral positions] are temporary and developmental*” [1]. This quote from the director of talent and skills at a major funding body in the United Kingdom (UK) succinctly highlights what postdoctoral positions are expected to be, but often are not. Linguistically, the inclusion of the prefix “post” in “postdoctoral” implies that such positions fall within a defined chronology, specifically of defined academic career stages. Within the traditional career progression pathway of an academic in a neoliberal university, postdoctoral positions are associated with the transitional career stage between PhD and lectureship [2], but how long is “too long” before this stage has exceeded its “*temporary and developmental*” phase is a matter of interpretation.

For the intergovernmental Organisation for Economic Co-operation and Development (OECD), it is when researchers reach “*an age at which they legitimately aspire to a more secured position*” [3]; what is legitimate at what age is undefined. Germany is, on the other hand, one of the only countries to have defined “too long”: researchers can only remain on fixed-term contracts for six years, beyond this, universities have to offer them permanent positions or to make them redundant [4]. In the UK, employees who have been in fixed-term employment for more than 4 years must be switched to open-ended contracts [5], but these can still include end-dates which coincide with the end of the funding (e.g. grant) period [see e.g. 6]. That way UK-based researchers need not be made redundant, but may stay indefinitely on contracts that are fixed-term in all but name. If we were to resort to proxies to know “how long is too long”, time limits on eligibility for early-career or postdoctoral independent fellowships may be good indicators: within 8 years of one’s PhD for the European Commission Marie Skłodowska-Curie postdoctoral fellowhips, within 5 years for the US Fulbright postdoctoral awards or for the Australian Discovery Early Career Researcher Award. Most opportunities in China both have age (up to 35 years old) and years since PhD (up to 3) restrictions. Conversely, most funders in the UK have lifted time limits for eligibility altogether.

It is also questionable whether this period is seen by all concerned as temporary or transitional. Most universities do not offer research-only career progression pathways (to the authors’ knowledge, only one in the UK does [7]), but 34% of research staff have been on temporary contracts for more than ten years at one or more Higher Education institution, including 13% who have been in such long-term employment at their current institution [8]. Thirty-eight percent of researchers in the UK also covet research-only positions [8]. Are we to assume that all these researchers and their employers see these positions as transitional?

The name of this transitionary period is also changeable. Researchers will take on many identities depending on the agenda of the authors discussing them. They can be defined by their place within the academic career chronology (postdoctoral, early career, junior or mid-career researchers—with the first three often used interchangeably), by their employment situation (“staff on fixed-term contracts” [e.g. 9]; “contract researchers” [e.g. 10]; “permadocs” for “permanent postdocs” meaning “forever postdocs” rather than “postdocs on permanent contracts” [11]), by more interpretative denominations as in “casualised” or “precarious” staff (e.g. [12]) or by more general terms which themselves reflect the lack of definition e.g. “academic related staff” (e.g. [13]), “contingent faculty” or “non-tenure-track-faculty” [e.g. 14]. That no single term exists to describe this population is in itself significant; it is hard to understand or to describe anything for which no word exists.

Failing widely accepted definitions and terms for these “researchers”, we must provide our own. We opted for a descriptive term that none of the participants of the study we present below explicitly objected to and that reflects the heterogeneity of the careers among participants: “long-term research staff” (LTRS). Secondly, we defined “long-term” as a minimum of 8 years post-PhD or highest degree in employment on research-only temporary contracts. Although this time length is beyond the upper limit of some of the fellowships listed above, we found it most appropriate within the UK context, where this study is conducted; up until recently, eligibility for most early career or postdoctoral independent fellowships were capped at 8 years after PhD. Finally, some LTRS are on fixed-term contracts, others are on the open-ended with end-dates contracts described above; whatever the terms used on the contracts, they are all temporary. For clarity all subsequent uses of the word “permanent” mean “designed to continue or last indefinitely” [15] and is therefore used in opposition to temporary within the context of short or fixed-term contracts.

Sisyphus was condemned to roll a boulder up a hill for eternity; every time he reached the top of the hill, the boulder rolled down again and he had to start all over again. For many LTRS, having to “start all over again” and permanently doing something that is temporary are familiar situations. This study is, to our knowledge, the first to be dedicated to staff in long-term employment on temporary research contracts; many have been dedicated to investigating different aspects of the lives and careers of staff on temporary research or teaching contracts [eg 16–19], but none have specifically investigated or isolated cases of those in long-term employment. Camus [20] hypothesised that Sisyphus found satisfaction in a situation others may find absurd; we will hypothesise the same of LTRS. The aim of this study is therefore to understand how and why some researchers—despite a rise in the number of postdocs that fuels competition for every advertised post [3, 10]—stay in long-term employment in positions that the academic career structure defines as temporary? To this end, our objectives are to understand 1/ the circumstances behind the career paths of research staff in long-term employment on short-term contracts 2/ the career motivations of long-term research staff (LTRS) 3/ the specific needs of LTRS in order to provide them, if necessary, with more institutional support.

## 2. Study design and methodology

The study design, methodology and participant information sheet (PIS; S1 File) were approved by written consent by the Moray House School of Education and Sport Ethics Committee at the University of Edinburgh, where the authors of this papers are based.

Following Lincoln’s [21] advice to researchers to “come clean” about their position on their research topic and in line with many protocols for reporting qualitative research [e.g. 22, 23], I (the first author and study lead; “we” hereafter refers to the two authors) declare that I am a long-term researcher. We also declare that, over the years, we had both been, separately, in conversations in which long-term research staff had casually been referred to as passive postdocs e.g. “floating” or “hanging on” to their PI. Coming from a researcher development perspective, the second author felt that we knew little about the type of training and support that LTRS may need or that could be offered by universities. Our respective experiences, which did not reflect this passive postdoc narrative, was critical when designing the study.

Having no prior information about the circumstances behind the career paths of LTRS, we decided that a case study would be the most appropriate method to give a voice to a community that, from our own experience, was misrepresented and had, so far, not been investigated. The aim and objectives stated in the previous section conform to one of the purposes of the case study, namely to provide descriptions of a group [24] and, rather than to test a hypothesis, to capture information about the how, why and what [25] of that group. As the interviews progressed, the case studies format also allowed us to generate a hypothesis [24].

The research was conducted in a single research-intensive university in the UK. As is shown in Section 3, many study participants had worked in multiple universities over their career, both in the UK and abroad; as such, we believe that their stories are not representative of the university in which the research was conducted, but more broadly of researchers in long-term employment on temporary contracts, albeit with a bias towards researchers in the UK or similar Higher Education systems. Data provided by Human Resources at this university showed that 10% of its research staff had been employed on temporary research contracts for more than eight continuous years, but less than 5% of these were employed in the Arts, Humanities or Social Sciences (HASS). We chose not to recruit HASS participants as we were concerned that any issues arising from their field of studies may make individuals more recognisable due to their low number.

The call for interview participants was distributed via a newsletter sent to all research staff, key School contacts and online research staff societies. Participants were asked to commit up to 90 minutes for the interview. Our initial target was 20 participants; 38 research staff contacted the study lead, 25 of whom agreed to being interviewed after being sent the PIS. No eligible volunteer was rejected. One interviewee did not correspond to the profile described in the PIS (they were a research manager, not a research staff) so their information was not used. No reason was given by any of the thirteen researchers who did not follow-up after being sent the PIS; I did not contact them further as I did not want them to feel pressured into participating.

The interviews were conducted following the protocol described below, in line with methodologies for semi-structured interviews [26, 27]. The participants were informed that the questions would revolve around the following themes, which captured information around the how and why of this study’s aim (Section 1):

- Working relationship with the research group and/or the principal investigator (PI i.e. the grant holder and line manager of the LTRS).
- Specific value and responsibilities in their research group.
- Is being a long-term research staff on fixed-term contracts deliberate or accidental/suffered?
- What is their career goal?
- Had they encountered obstacles or been discriminated against?
- Had they identified any training needs?

I conducted all the interviews, which took place on Microsoft Teams and Zoom between April and June 2021, when the Scottish Government advice with respect to COVID-19 was for office workers to work from home if they could. All interviews were video recorded and real-time transcription enabled. As the accuracy of transcripts depends on many elocutionary factors (e.g. speed of delivery, articulation, accent), I checked and corrected each of them within three weeks of the interview. In many cases, correcting the transcripts took longer than the interview themselves, but it meant that I listened to all the interviews in their entirety a second time and quickly gained familiarity with within-case data.

The transcripts were referred to throughout the redaction of this paper and were checked constantly to ensure accuracy of the reported stories. I am the only one to know the identity of the participants and to have access to the transcripts. As such, it was important to offer the participants the opportunity to object or to confirm my interpretation of their story: prior to submitting this research for publication I gave a talk describing the research findings to which they were invited and also provided them with the opportunity to read an earlier draft of this manuscript. No participant objected to the findings presented here.

I began all interviews by telling the participant that they could refuse to answer any question. I stressed that, to collect robust data, it would be preferable not to answer than answer partly or hold information to avoid any misinterpretation from my part. Only one participant chose not to answer one question. A few times, to protect their confidentiality participants asked me not to relay specific details; a few other times in the rest of this paper, we decided to sacrifice relevant information for the same reason.

I then asked the participant to describe their professional journey since their PhD or undergraduate degree, whichever was last. All themes listed above were addressed as and when it felt most appropriate during the interview. Themes that had not been listed and similarity between stories told by participants emerged quickly e.g. regarding access to and recognition around teaching. Other themes that had been anticipated (e.g. discrimination around some protected characteristics) could not be verified because of the demographics of the participants (described below). When an additional topic was discussed, I was clear that this new theme had emerged from discussions with other participants.

## 3. Demographics of participants

**Fig 1:**
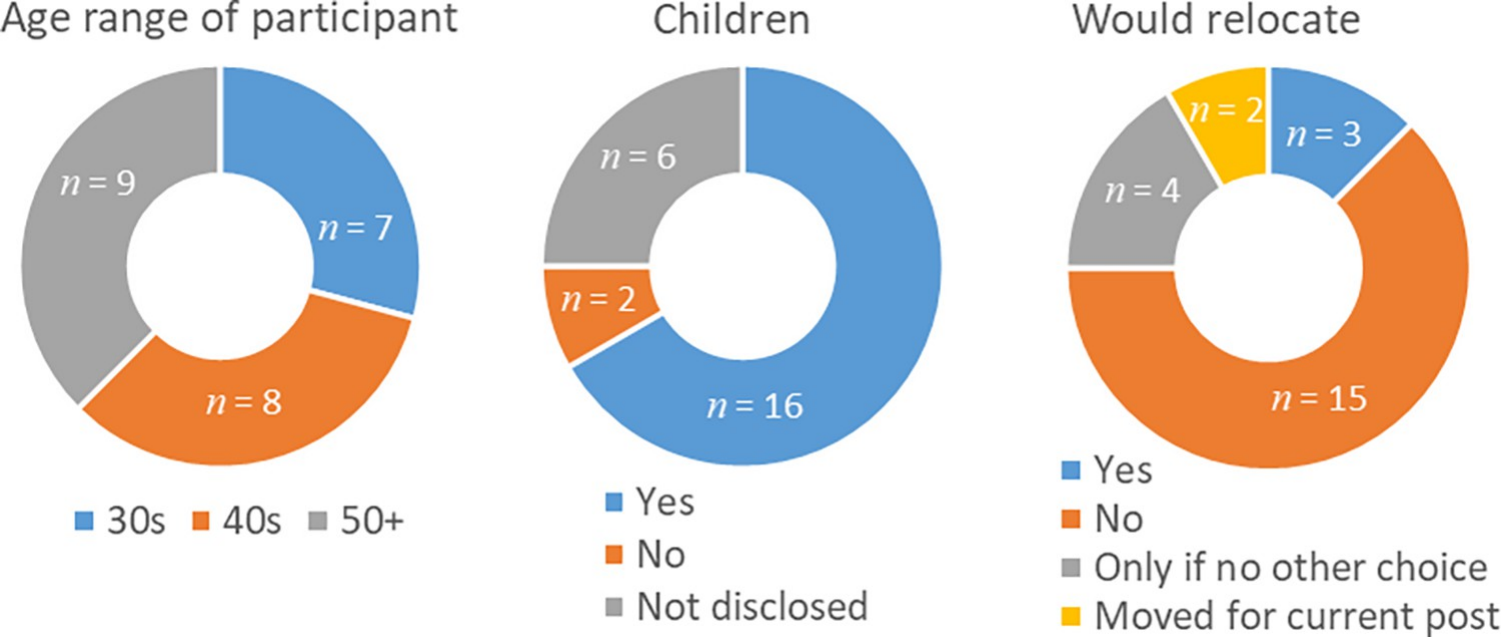
Demographic information about the research participants. Left: Disclosed and estimated age in 10–year age bands. Middle: Number of participants with children or otherwise. Right: Willingness to relocate for a position.

Of the information shown in Fig 1, the participants were asked only about their willingness to relocate. For most, the answer was related to their age and family situation, both of which were information that most interviewees volunteered. When it was not the case, the age of the participants was estimated within 10-year age bands. At least two thirds of participants had children.

**Fig 2:**
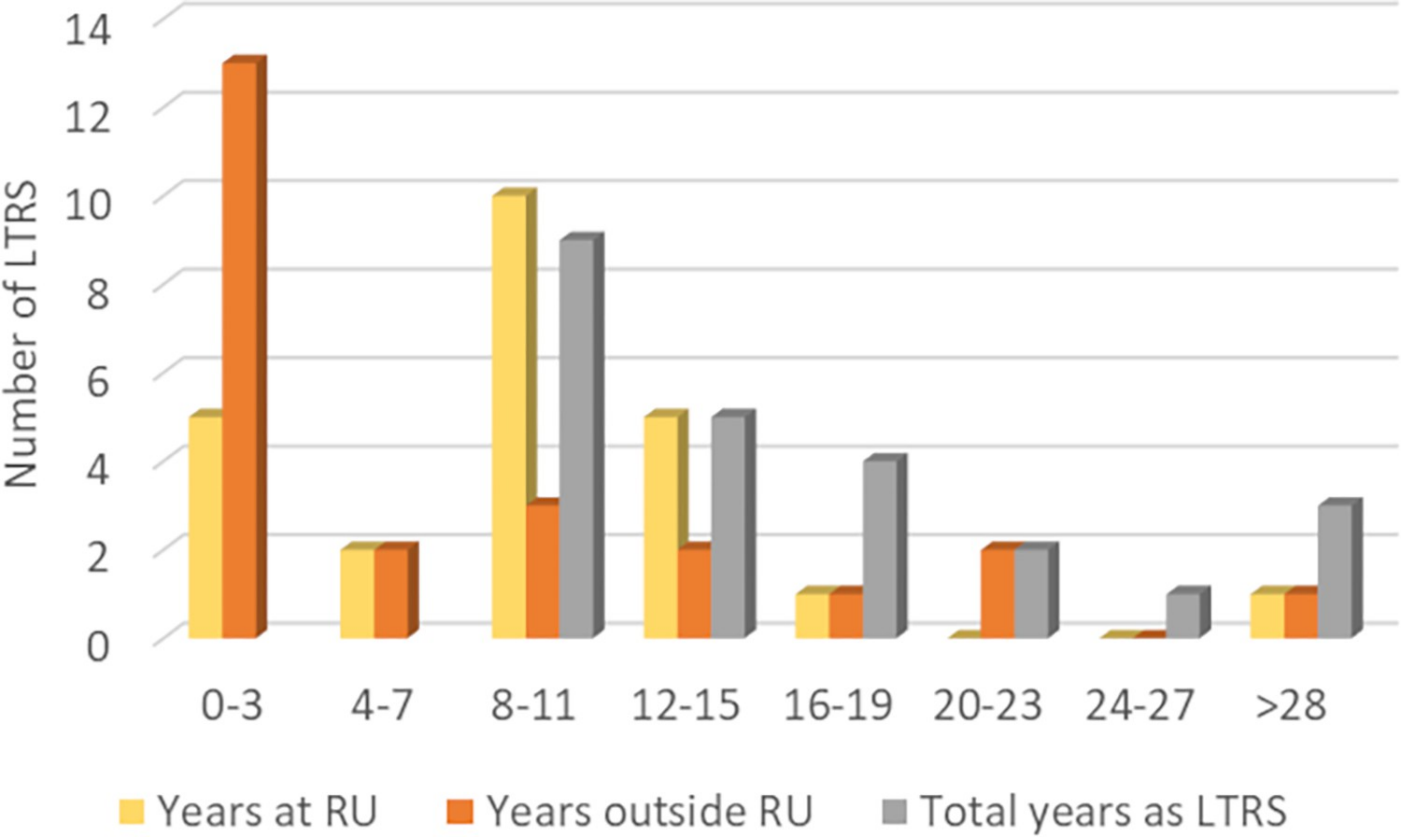
Number of years the study participants worked outside and at the research university (RU) where this study was conducted and total number of years as a long–term research staff. Note that the grey bars add up to the number of participants (24), but that the yellow and orange bars do not because some participants have worked both outside and at the RU.

The number of participants was inversely correlated to their number of years as LTRS (Fig 2). In the absence of sector-wide data, numbers in Fig 2 can only be compared to data of LTRS employed continuously for more than 8 years at the university where the participants were based, where the number of LTRS decreases by 63% between 8–11 years and 12–15 years of service and by a further 58% years for 16–19 years. Although these numbers do not correspond to our population sample (50% between 8–11 and 12–15 years, and 80% between 12–15 and 16–19 years), both data sets suggest decreasing number of LTRS with increasing years of service. Fourteen women (58% against 50% for all research staff at this university) and 10 men participated. Only seven participants (29%) declared not being British-born, against almost double this percentage (56%) for all research staff at this university. Although we can only speculate at this stage as to why there is such a difference, it is possible that fewer research staff who require a work visa do or can remain in the UK long enough to become LTRS. Two thirds of the participants were on Grade 7, the most common pay grade for postdoctoral researchers and one third was on Grade 8, the grade generally–but not always–associated with senior researchers and lecturers at this university who, unlike LTRS, are in a permanent position. All but two participants had a PhD. No participant disclosed being LGBTIAQ+ or BAME. One participant disclosed having a disability.

In order to protect their confidentiality, all participants are referred to with gender-neutral pronouns unless the disclosure of gender is relevant to the findings. Hereafter, the term “academic” is used to describe staff on a teaching and research contract in a permanent position; this conventionally includes positions from assistant-professors or lecturers to professors.

## 4. Results

### 4.1. Categories of long-term research staff

One of the particularities of interviews as a data collection method is that data analysis occurs concurrently with collection [26]. Before we started the interviews, we had expected the outcomes of this study to be organised as per the themes addressed during the interview and described in Section 2. However, as the interviews progressed, it emerged that the themes were addressed and had been experienced differently depending on where the participants situated themselves within the traditional career structure offered by universities (PhD–postdoc–lecturer up to professor): whether they wanted to become PIs, whether they did not–decision for which reasons varied -, whether they had set themselves career goals or not, etc. Based on this, we hypothesise that three categories of long-term research staff, which we describe in the next sections, exist: “the candidate”, “the accidental LTRS” and “the career researcher”. These categories are neither mutually exclusive nor sequential and most participants had belonged to at least two throughout their career.

**Fig 3:**
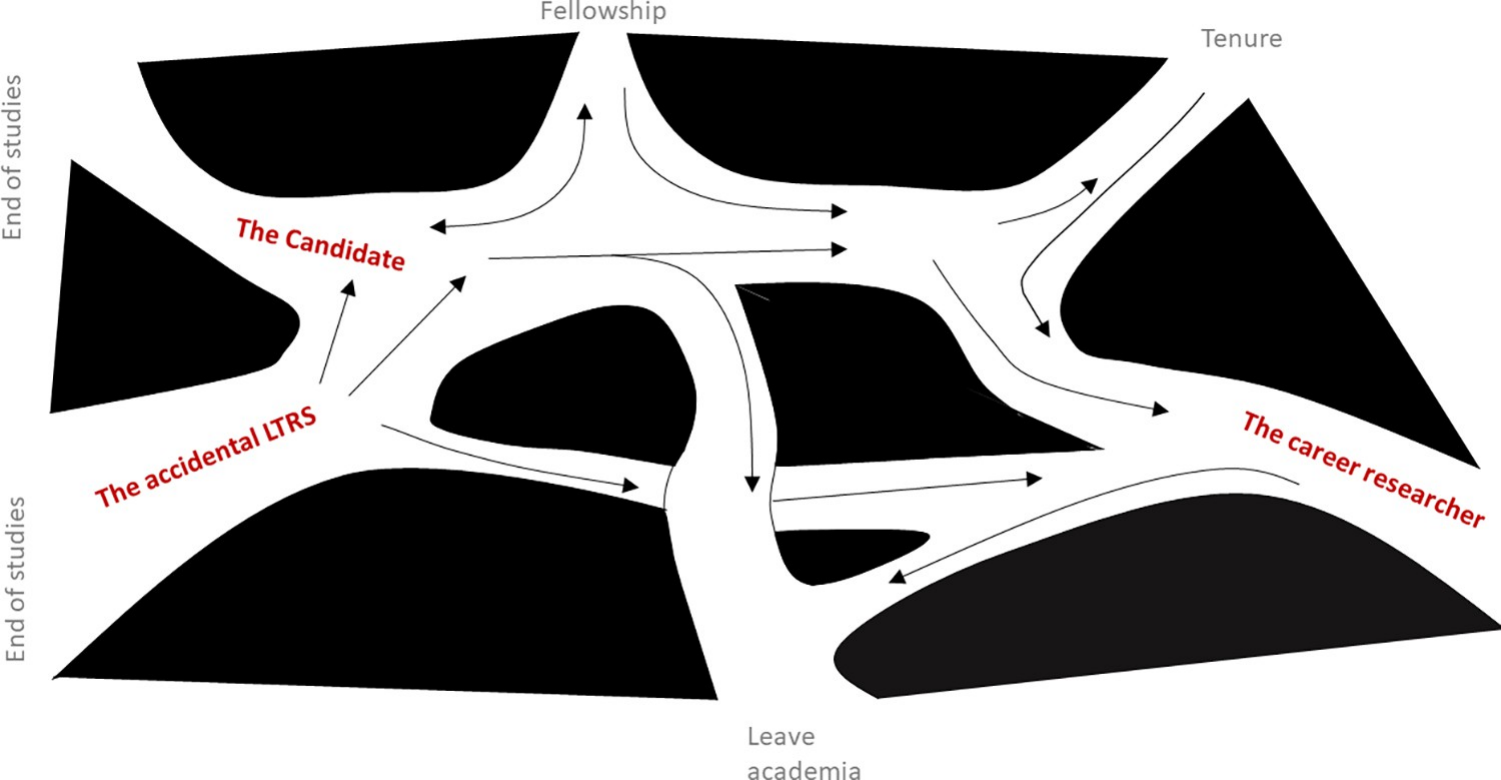
Schematic representation of the career paths of LTRS. Each arrow represents a path taken by at least one of the participants.

The title of this study as presented in the information sheet distributed to the participants was “Long-term research staff: deliberate or accidental career choice”; some participants pointed out that their career path had been a combination of the two. OECD [2] identified that there was a cultural expectation in academia that careers followed linear paths from doctoral students to junior researcher to lecturer, all the way up to professor. Fig 3 represents the career paths that this study’s participants have taken. Each arrow represents a path taken by at least one of them, paths that are discussed in Sections 4.1.1. to 4.1.3; the authors will let the readers decide whether they fit their definition of “linear”. As already mentioned, sector-wide data of LTRS are inexistent; these paths, based on 24 self-selected volunteers only, are unlikely to be exhaustive, but they are at least representative of the participants rather than being speculative of other possible paths. We do note that approximately 12% of “postdocs” in the UK become academics [28] and that 13% of our participants had.

#### 4.1.1. The candidate

The “candidate” is the researcher who wants to follow the traditional academic career pathway and, thus, applies for the chronological next step to postdoctoral positions i.e. for independent research fellowships and lectureships. Twelve participants had, at some point in their career, been in this category. Half of them became candidates straight after obtaining their PhD, half had first been accidental LTRS. Two in the latter group and one in the former were now in academic positions.

Research, teaching and management are the three pillars of the modern academic [e.g 29, 30]; the determining feature of the candidate is that they actively seek opportunities to build their portfolio accordingly. Funding bodies generally do not allow staff on fixed-term contracts to be principal investigators on research grants so one’s ability to secure funding is best proven by securing an independent fellowship; being a co-investigator on a grant does not carry as much weight. As such, of the twelve candidates, all had applied for independent fellowships, five of whom had, at some point, secured one. Although fellowships are commonly believed to be springboards to securing an academic position, one of the candidates had, so far, failed to follow-up their prestigious fellowship with another one or with a lectureship. Four candidates perceived their publication record to be the main obstacle to them securing a lectureship: they either had no high impact publication or not enough papers as first author. On the other hand, one of the candidates now in an academic position conjectured that their publication list had been essential to secure their position: “*I probably have more publications than quite a lot of my contemporaries who have had permanent contracts for quite a few years*”.

This candidate was the only one not to have had extensive experience in teaching. All others had proactively (by definition the type of “research-only” contracts the participants are employed on do not include teaching) sought out opportunities to develop this side of their portfolio. Two candidates had gained a teaching accreditation awarded by the Higher Education Academy and all but one had taught on undergraduate courses and / or co-supervised PhD students.

Opportunities to prove management or leadership skills also needed to be actively sought out. Some candidates had sat on various Schools or Colleges committees or co-supervised early career researchers. A few had experience in research project management and two, independently of their PI, had been involved in international or government reports for policy-makers in their respective disciplines.

The candidate was the most likely to express their intention to leave academia if they did not secure an academic position soon “*I’m at the point that if a good opportunity elsewhere arises*, *I will probably take it*. *I’m bored and I’m sick of it*”. Two of the five participants who moved from accidental LTRS to candidate affirmed that their current post would be their last one. While they both expressed being fed up with their precarious employment situation, they also condemned the effect of short-termism on their career and motivation: “*working even just four hours a week at times was fine when it was serving a [research] purpose*. *But to continue doing this now from month to month without actually having a goal or reason why I’m doing this feels pointless*”. Of all participants, the candidates expressed the most being dissatisfied with their situation and with the lack of commitment of their institution towards them i.e. with the institution’s failure to offer them a permanent contract despite their contributions to the success of their School or Institute. This echoes one study that showed that, in academic careers, the greater the discrepancy between present job and desired job, the more negative attitudes were [31]. We speculate that this frustration was exacerbated by the number of years, sometimes up to seventeen, candidates had been working towards and applying for these positions.

#### 4.1.2. The accidental long-term researcher

The accidental long-term researcher is a researcher who did not or could not plan their career path. For those who did not have a career plan, the “accidental” career pathway generally took a welcomed turn, which led them to be continuously employed. For the others, the accidental nature was generally suffered i.e. systemic obstacles prevented them from fulfilling their career plans. The specifics of each group are detailed below.

Group 1: “The successful research group” accidental LTRS. Unlike accidental LTRS in the three other groups, members of this group usually had worked in the same research group or with the same research lead (e.g. PI) for most of their career so far, sometimes since their PhD; what the research group or PI are “successful” at is securing funding. These LTRS generally work on multiple (simultaneous and /or successive) research projects on which they were named since the proposal stage. For the research group, employing a researcher they already know and who has the required skills is about minimising risk. For the researcher, it is generally a job in a research area they love and, for some, it is working in a research group they know well and in which they have an already established, albeit contractually precarious, position. It can also be the best group in their field: ““*My [research speciality] (…) has always been very big in the institute*, *(…) that’s the reason I came here to start with*. *Even if I didn’t have [to stay in this city because of my] kids*, *if I could choose wisely*, *I would still prefer to be here*”.

Group 2: “The no PhD” accidental LTRS. Two of the participants did not hold a PhD. Although they described this as being a hurdle to their career, their job title, grade and many of the issues that they had encountered were, in fact, the same as the other participants. Nevertheless, opportunities *had* been fewer because they had never been eligible to apply for fellowships or lectureships. They admitted often taking positions because they needed the money rather than having been able to secure positions—unlike many other participants—that would have allowed them to develop expertise in a chosen field. That universities do not offer a career progression pathway for research-only staff was deemed regrettable and was partly thought to be the reason behind the heterogeneity of their own career path.

Group 3: “The interdisciplinary” accidental LTRS. Four of the five participants who described themselves as interdisciplinary said that they “did not fit the box”. Those who had never applied for a lectureship and/or fellowship had not done so because advertised positions or schemes had been too discipline-specific. Those who had applied found the reviewing process inadequate; even when they had applied to schemes open to interdisciplinary researchers, they argued that reviewers themselves had been too discipline-specific and had not had the relevant expertise to review elements in the proposal outside their own discipline. For lectureships, one participant described having been interviewed for positions in the three disciplines relevant to their research, but was told each time that they had not been specialised enough.

Group 4: “The part-time” accidental LTRS. One group of participants stood out above all others by the similarity of their stories: part-time researchers, of all whom were women and all of whom but one had opted for this work pattern after having had children. Extensive literature about the relationship between the gender gap in academia, childcare and part-time work exists, but fewer papers focus on research staff specifically [e.g. 32, 33]. The seven part-time participants all said that the perceptions around part-time work had in the long-term been the biggest obstacle to their chosen career pathway “*The way full time people view part-time work is almost as if the work you do is not good because you’re not doing it full time”;* “*I feel that as soon as you say to people you work part time*, *they’re dismissive*, *as if you’re not serious about your research career*, *which I find personally quite offensive because you can be just as dedicated to research*, *but just choose to do it part time*”; “s*ome [colleagues] make you feel like* ‘*Yeah*, *she’s only part time’*, *she’s got to get the kids from school*’ “. While perceptions are not obstacles in themselves, they are when mismanagement of part-time staff and misconceptions about what part-time work entails lead to unequal opportunities. “*I didn’t ask [the PI] until I had got offered the job if I could work part time*. *[The PI] said to me*, *do you think you can manage to do that in the part-time hours*? *I didn’t understand at the time what [they were] asking*, *but I think [they] generally thought that it was a full-time job at part-time hours*. *[They thought that if] you were working part-time you were doing your job over compressed hours*, *but that certainly wasn’t how I saw it*”. “*In terms of your CV over the same amount of time you haven’t achieved the same amount of publications [as full-time colleagues]*. *Although people do supposedly take that into account*, *I think it’s hard for them to see that it’s equivalent*. *I think there is some discrimination or some sort of unconscious bias*.” “*I think people don’t know how to develop you*. *(…) My boss doesn’t give me extra tasks to do like be involved in writing grants because they’re trying to be respectful of my time in terms of me not working full-time*, *but that means I’m not getting to do career development tasks*.” It is worth noting that the participants who had returned full-time after their maternity leave had done so partly because they had been told that they would never progress in their career on part-time hours. While many funding bodies now allow for career breaks to extend eligibility for independent fellowships, this was not always the case; eligibility criteria for participants in this group had, in the past, closed the opportunities and provided barriers to their career progression.

#### 4.1.3. The career researcher

Eleven participants were career researchers. Eight had started as accidental researchers, two had been academics who left their position because of changes in circumstances in their personal life and just one had been a candidate. The line between the accidental and career LTRS is blurry, but we trace it at the point in the researcher’s career when they conscientiously decide to pursue a career in research although they know that the university sector does not generally support research-only career pathways. “*I began to realize over the years that something always worked out so I just kind of stopped worrying about [my next job]*”.

There were two types of career researcher among the participants. The first type is an extension to the first group of accidental researchers: they have in-depth and unique knowledge in one of the tools of their trade (e.g. instrument, model), they contribute to research proposals–sometimes lead them, but can not be named as PI because many funders require PIs to be in permanent positions-, they publish, and they can teach and supervise PhD students and junior postdocs. They are *invaluable* to their research group in the etymological sense of the word: the financial and temporal costs of replacing them—if at all possible—is not quantifiable. The second type is the career researcher who is adaptable to the requirement of a range of projects and whose skills and intuition are transferable across disciplines; it seems that interdisciplinarity, unlike for fellowships and lectureships, is an asset when it comes to becoming a career researcher. This type has worked in multiple institutions and their reputation and extensive network have been a reliable source of job positions: “*I have a really good network of colleagues (…) I have a reputation for being good in my field and so I’ve built that up”*.

No career pathway starts with being a career researcher for the simple reason that the overwhelming majority of universities do not propose career pathways leading to a research-only career. Yet, half of the career researchers had been LTRS for more than 20 years meaning that, against all odds, they had maintained a career on a path that, in theory, does not exist. When asked about their endurance, the participants invariably quoted their years of experience: “*There’s a huge amount of skill that you just accumulate through the years that is not easy to describe on a CV*. *PIs get a lot of expertise when they employ more experienced postdocs*: *we have done it before*, *we’ve seen what went wrong and we know what made it happen*. *We’ve accumulated a huge amount of intuitive troubleshooting*.”

Seven career researchers had never applied for a fellowship or lectureship, either because of lack of opportunity (see Section 0) or because they did not believe that the grass was greener on the other side: “*one of the major drawbacks of going into a lectureship position is it really is two jobs*. *(…) There are a lot of negatives to staying a postdoc*, *but at least the work-life balance is quite good*.” The high level of stress amongst academics [34–36], and, in some disciplines, the growth in large scientific teams led by a few select leaders supported by scientists in specialized roles [37, 38] are some of the reasons why the career researchers found research-only positions more attractive and attainable despite their precariousness.

### 4.2. Cross-category issues

#### 4.2.1. Managerial skills of PIs

The single most cited stroke of (bad) luck that influenced the participants’ career paths was their line manager. For better or worse, the career path of many participants had shifted because of the support or otherwise of their PI. Five participants explicitly quoted “*it’s who you know*” as the main criteria to secure a permanent position–including the two who had recently done so internally; more hinted at it. “*Internal people are not valued as much as they should*, *unless they are with a PI who somehow aligns with the system*. *It feels almost like permanent positions are given to some research staff as a reward to the PI*.*”*

Some participants also stressed the need for PIs to be trained on how to develop their research staff, how to manage research staff at different stages of their career, how to manage part-time staff, etc. Four participants said that only after changing research group, which they had done recently, had they realized that their previous research group had not provided development opportunities. For three of them, this lack of realisation had been partly fostered by the fact that their line manager had been a “*nice*” person: “*My previous PI was supportive as a human being*, *but*, *[retrospectively*, *I think] they weren’t the best at knowing the system*. *They were quite old fashioned*”. Good interpersonal relationships had hidden poor managerial skills.

#### 4.2.2. Bullying and discrimination

Wanting to remain with a friendly PI is understandable when the majority of researchers in postdoctoral positions report having experienced (65%) and observed (74%) bullying [41]. In addition, a large minority (from 24 to 43%) reports having experienced or observed gender and racial discrimination, and sexual harassment. In this study, three participants declared having been bullied in a previous position; one more described a “*conflict*” with their research lead. One form of bullying–exclusion—which was experienced by two participants, is a covert one and is therefore not always recognised as such, even by the victims: “*I was totally overlooked for any sort of career development opportunities […] I don’t know if I’d call that discrimination or just bad management”*. The percentage of participants who had declared having experienced bullying (25%) was lower than reported in Woolston [39] or in the Wellcome research culture survey [40]. This may be explained by the fact that just under one third of this study’s participants had worked for more than eight years in the same research group; such a long working relationship implies at best, a good, and at worst, a bearable working environment. Unsurprisingly, none of the participants who had experienced bullying were in this group.

Discrimination had been experienced by 25% (*n* = 6) of participants. One participant believed that they had been a victim of ageism when applying for, but not securing, postdoctoral positions in their 50s. Female participants were they only one to mention having experienced gender discrimination (*n* = 5). Manifestations of this discrimination varied, from being taken less seriously in a male-dominated field, to being demoted to a less prominent place than their male colleague in a project proposal or to being the “part-time mother” discussed in Section 0. When asked whether she had ever been discriminated against, one participant answered that she had not, but later in the conversation mentioned how differently she had been treated after she returned, full-time, from maternity leave. When I highlighted that she was describing discriminatory behaviours, the participant answered: “*Discrimination against women is so widespread that I didn’t think it was necessary to highlight*”.

#### 4.2.3. Teaching

For staff on research-only contracts, being allowed opportunities to gain teaching experience depends on the good will of their PI. “*I asked about support for time to develop teaching skills for postdocs*. *I got shut down*. *I was told that there was a tension between teaching and what I was being funded to do*”; four other participants reported similar positions from their line managers. One of the participants had been told by an academic manager that “*teaching evidence [was] not essential for applying to [research] universities*”, another, whose teaching portfolio was more extensive than their publication record, was told that their CV would be more appropriate for a “*minor*” university. Lacking teaching experience is particularly detrimental to LTRS in the candidate category who may apply for lecturing positions against candidates with extensive teaching portfolios.

For those who are allowed to teach, recognition of the work can be problematic. At many universities, named course organisers or leads must be academic staff even if most of the work is done by research staff. One third of participants mentioned having supervised PhD students, but most had not been officially recognised as a co-supervisor. Guaranteeing continuous supervision to students when on temporary contracts is also problematic: *“[Once I applied for a position] and they said I didn’t have enough teaching experience and I didn’t have enough PhD supervision experience*. *(…) How are you supposed to supervise students [for a 4-year] PhD when you only have short-term funding*?”

#### 4.2.4. Prestige of research field

Three participants said that their area of expertise was “*not sexy enough*” to warrant publications in high impact journals, thus not making them competitive for academic appointments. On the other hand, three participants—including the two with the longest employment on short-term contracts—said they had been lucky that the field they had worked in had been emerging at the start of their career and had expanded almost beyond expectation. We argue that their longevity as career researchers may not owe strictly to this early stroke of luck, but rather to their expert skills and knowledge whose demands increased over time.

## 5. Discussion and recommendations

As mentioned in Section 2, this study was motivated by conversations the two authors had been in and in which long-term research staff had been represented as passive postdocs “hanging on” to their successful PI. Our findings show a rather different story. Nevertheless, the “passive postdoc” narrative is convenient to justify systemic shortfalls in neoliberalist universities. At its root, the bottleneck experienced by researchers, long-term or otherwise, is simple and oft explained: “tenure is dying” [41]. Universities alone are not responsible for the dearth of permanent positions. In the UK the short-term cycle upon which university funding allocations are distributed—which is aligned with the currently 6-yearly Research Excellence Framework (REF) [42]–has been accused of being responsible for short-termism in Higher Education [e.g. 43, 44]. Concurrently, the number of doctorates is rising [45–47] which causes higher competition for postdoctoral positions. If postdocs stay in the system for longer than an “acceptable” transitional period (the shifting length of which is discussed in the Introduction) the market becomes saturated with the arrival of new doctorates. At the other end of the road, the lifting of a compulsory retirement age in the UK in 2011 also contributed to the bottleneck becoming narrower.

Rather than the circumstances encountered by the participants being specific to the university in which we conducted this study—many had spent part of their career at other institutions—we argue that they rather reflect a systemic failure: the traditional academic career structure has not evolve at the same pace as the demographics and practices of the scientific community. There have been many studies on postdocs and early-career researchers, but, to our knowledge, this is the first study dedicated to long-term research staff. So where do we go from here?

Firstly, future work should aim to upscale our findings from case study at a single institution to a national, or at the very least multiple-institution, investigation. Not only do we not know the percentage of research staff that LTRS represent in UK Higher Education (the numbers provided in the Introduction are based on a survey, not on sector-wide data [8]) let alone internationally, but we knew nothing of the circumstances behind their individual career path. The numbers had been assumed to be small and the paths similar. This case study shows that, at one research university in the UK, at least 10% of research staff are LTRS and that there is no set path; given the limitations of our small sample of participants, which, for example, lacked representation of some groups with protected characteristics (e.g. ethnic minorities, LGBTIQ+), more categories and paths may emerge.

Secondly, further work should also investigate the case for long-term researchers from a stakeholder’s perspective. Why do PIs employ LTRS? Why do many funders restrict eligibility criteria such that PIs must be in permanent positions, thus contributing to the invisibility of LTRS? The quote that starts this paper implies that long-term researchers exist because of bad managerial practice: supervisors should not employ researchers beyond the postdoctoral “transitional and developmental” period but do. As our findings show, the reality is somewhat more complex. For example, with the support and encouragement of their PIs, one third of accidental LTRS had moved to the “candidate” category; they did not fit the passive postdoc nor did their PI fit the bad supervisor narrative but they were still, nonetheless, LTRS.

One of the participants pointed out that the field they were in needed large research groups to address their scientific questions. Indeed, the research group has replaced the individual scholar in publications, as the increase in the number of authors per publication shows [48], and become an important measure unit in exercises that quantify research quality such as the REF [49, 50]. However the growth of large research groups and consortia, which is encouraged by funders, has fostered a pyramidal labour market where the one research group leader is supported by many staff on precarious contracts [51].

Our data suggest that LTRS are often critical to the success of their research group or even are the instruments of their own success, but systemic shortcomings–e.g. eligibility restrictions in funding applications, absence of a promotional pathway for research-only staff—mean that their contribution to the financial success of neoliberal universities are played down. On the other hand, not all scientists can or want to be group leaders or PIs [52]. Many of the accidental LTRS described themselves as having had “no career plan” at the start of their career. For those whose aim would have been to be a career researcher (after all, eight now are), the impression of having had no career plan could have been fuelled by sustainable career progression pathways not being proposed by their institution. After all, “you can’t be what you can’t see” (Marian Wright Edelman [53]). In addition, the classic career progression pathway (PhD–postdoc–lecturer) fails to recognise that research skills are different from teaching skills; is there not a dichotomy between training PhD students and postdocs to be researchers and not providing stable career options in research-only positions?

This leads to the final recommendation. Our study shows that research-only career pathways are diverse and further research should investigate how best to represent and to support this diversity rather than to impose the unique and misrepresentative narrative of so-called postdoc positions being”*temporary and developmental*”. Based on the career paths of our participants, we anticipate that one of the outcomes should be for universities to propose a transparent framework for progression in research-only careers. More broadly, we recommend that the HE sector review the academic career structure and that a cost-benefit analysis of supporting a career model with higher number of research staff in permanent positions be conducted alongside the review. The cost of shifting to such a model is often considered to be prohibitive [54, 55], but, to our knowledge, no study has yet investigated if the higher financial risk that universities would take by employing more staff permanently may, in fact, be offset by the higher number of staff being eligible to apply for, and by extension to secure, funding.

## 6. Conclusion

We believe that this paper is the first to examine the career pathways, or even to give a voice, to research staff in long-term employment on short-term contracts. Based on semi-structured interviews with 24 LTRS from one university in the UK, we hypothesise that the careers of long-term researchers shift between three categories: 1/ the candidate, who has built or is building a portfolio in view of securing a position on the next step of the traditional academic career progression pathway 2/ the career researcher, who has made a conscious decision to sustain a research-only career despite the precarity of such positions 3/ the accidental long-term researcher, who did or could not, because of systemic obstacles, plan their career path. We also found that some obstacles to career progression—such as poor or inexistent managerial support, unequal access to opportunities for developing one’s teaching portfolio, the perceived prestige or lack thereof of one’s field, and bullying and discrimination—transcended the categories.

We argue that this study provides the first insights into a potentially large, but, up until now invisible and invisibilised, part of the research community and should provide the grounds for further research. The aim of this study was to understand how and why some researchers stay in long-term employment in positions that the academic career structure defines as temporary. Answering why is straightforward and sounds almost too simplistic: some want to lead research, others are happier to conduct research driven by others, but they *all* want to do research. The “how” is also simple: LTRS contribute to the research and financial success of their institution. The complication with the “how” is that these contributions remain unrecognized, or at least are played down, because of various systemic obstacles (e.g. academic career structure, eligibility for funding opportunities). We therefore recommend that future work engage with multiple actors in the HE sector (other LTRS, universities and funders). Of particular importance, we encourage future research to investigate whether the economic benefits of supporting research-only career pathways, may, in fact, outweigh the costs of staying in what our research suggests is a career structure ill-adapted to the needs and the demographics of contemporary research.

## Supporting information

S1 File Participant information sheet. Information that would allow the identification of the university where the participants were employed was modified i.e. “[university name]” appears instead of the actual name of the university.
(PDF)
