# Supplementary material for: The career paths of researchers in long-term employment on short-term contracts: Case study from a UK university
Source: PLoS One. 2022 Sep 9;17(9):e0274486. doi: 10.1371/journal.pone.0274486 (PMC9462711; doi:10.1371/journal.pone.0274486)
Supplement: S1 File — Information that would allow the identification of the university where the participants were employed was modified i.e. “[university name]” appears instead of the actual name of the university. (PDF) [file pone.0274486.s001.pdf]

PARTICIPANT INFORMATION SHEET

Long-term research staff: deliberate or accidental career choice

You are being invited to take part in research on "Long-term employment amongst research staff on short-term contracts". Dr. Cecile Menard, IAD secondee, at the University of Edinburgh is leading this research. Before you decide to take part it is important you understand why the research is being conducted and what it will involve. Please take time to read the following information carefully.

<u>WHAT IS THE PURPOSE OF THE STUDY?</u>

The overall aim of the research project is to reduce precarity among research staff at [university name].The purpose of this specific study is to understand the circumstances and career path that lead to long-term (8+ years) employment amongst research staff on fixed-term contracts.

Precarity is linked to successive short-term positions and is a growing concern in Higher Education institutions. Although the number of research staff positions has increased at a much higher rate than the number of permanent academic positions in the past few years, funders and HE institutions still insist that these roles should be seen as temporary and developmental. However, based on a small number of interviews conducted with long-term research staff and a Nature survey, we argue that part of the research staff population has no intention to "progress" to an academic career, but instead wish to remain research staff. In addition, previous studies have raised serious concerns about inequalities, most notably by highlighting the prevalence of women, working-class and non-native staff amongst precarious researchers.

We propose a series of semi-structured interviews to understand:

- The circumstances that lead to long-term employment on short-term contracts
- The career motivations of long-term research staff
- The impacts on researcher progression and employability

Questions will focus on the following themes:

- Years since PhD, length of contracts, number of successive posts
- Working relationship with PI and other group members
- Specific value and responsibilities in their research group
- Is being a long-term research staff deliberate or accidental/suffered?
- Had the interviewee always aimed for a career in research and were they aware of the small number of permanent positions?
- What is their career goal?

- Is there any support they wish the university would provide e.g training, workshops?
- Do they have suggestions for interventions or changes that would have positive impact on their career
- If the [university name] supported permanent research staff, would they be interested, even if it meant being moved from one group to another?

WHY HAVE I BEEN INVITED TO TAKE PART?

You are invited to participate in this study because you have been identified as a long-term research staff.

DO I HAVE TO TAKE PART?

No – it is entirely up to you. If you do decide to take part, you are still free to withdraw at any time and without giving a reason. Please note that your data may be used in the production of formal research outputs (e.g. journal articles, conference papers, theses and reports) prior to your withdrawal and so you are advised to contact the research team at the earliest opportunity should you wish to withdraw from the study.

If you do decide to take part, please keep this Information Sheet. You will be asked to sign an Informed Consent Form to show that you understand your rights in relation to the research, and that you are happy to participate.

WHAT WILL HAPPEN IF I DECIDE TO TAKE PART?

You will be asked a number of questions regarding your career path and the choices you made throughout your career. The interview will take place remotely on Microsoft Teams at a time that is convenient to you. Ideally, we would like to record the interview. The interview should take around 60 to 90 minutes.

WHAT ARE THE POSSIBLE BENEFITS OF TAKING PART?

There are no direct benefits, but by sharing your experiences with us, you will be helping Dr Cecile Menard, the Institute for Academic Development and the [university name] to understand better the career path, needs and aspirations of long-term research staff, and the relationship between employment on fixed-term contracts and precarity. There may be indirect benefits because (1) IAD may use the results of this study to establish the particular needs of long-term research staff and to provide more support (e.g. specific workshops, training) (2) In line with the Concordat to Support the Career Development of Researchers [university name] Action Plan, actions to reduce precarity among research staff are being considered. Findings from the research project may support the actions.

ARE THERE ANY RISKS OR DISADVANTAGES ASSOCIATED WITH TAKING PART?

There are no significant risks associated with participation.

WILL MY TAKING PART BE KEPT CONFIDENTIAL?

Your data will be processed in accordance with Data Protection Law (further information available https://www.ed.ac.uk/records-management/policy/data-protection). All information collected about you will be kept strictly confidential. Unless they are anonymised in our records, your data will be referred to by a unique participant number rather than by name. If you consent to being recorded, all recordings will be destroyed once they have been transcribed. Your data will only be viewed by the researcher. All electronic data will be stored on a password-protected computer file. Your consent information will be kept separately from your responses in order to minimise risk.

WHAT WILL HAPPEN WITH THE RESULTS OF THIS STUDY?

The results of this study may be summarised in published articles, reports and presentations. You will not be identifiable from any published results. Quotes or key findings will always be made anonymous in any formal outputs. Anonymised information may also be kept for future research. A summary of the findings from the study will be made available to participants who indicate they would like to receive this. This summary will be sent to participants by email.

WHO IS ORGANISING AND FUNDING THE RESEARCH?

This study has been organised by Dr Cecile Menard, IAD secondee, Dr Sara Shinton, Head of Researcher Development, IAD, and Nicola Cuthbert, Research Development Manager, IAD and supported by [university name].

The study is being funded by The Scottish Funding Council and supports the ['s commitment to the Concordat to Support the Career Development of Researchers

WHO HAS REVIEWED THE STUDY?

The study proposal has been reviewed by the School Ethics Committee.

WHO CAN I CONTACT?

If you have any further questions about the study, please contact the lead researcher, Dr Cecile Menard, cecile.menard@ed.ac.uk.

If you would like to discuss this study with someone independent of the study please contact the School Ethics Committee, cahss.res.ethics@ed.ac.uk.

If you wish to make a complaint about the study, please contact:
Sara Shinton, Head of Researcher Development, IAD, sara.shinton@ed.ac.uk or the Research Governance Team (cahss.res.ethics@ed.ac.uk)
